# Differences in the health transition patterns of migrants and non-migrants aged 50 and older in southern and western Europe (2004–2015)

**DOI:** 10.1186/s12916-018-1044-4

**Published:** 2018-04-23

**Authors:** Matias Reus-Pons, Clara H. Mulder, Eva U. B. Kibele, Fanny Janssen

**Affiliations:** 1 Population Research Centre, Faculty of Spatial Sciences, University of Groningen, Groningen, The Netherlands; 2 Interface Demography, Department of Sociology, Vrije Universiteit Brussel, Brussels, Belgium; 3 Statistical Office Bremen, Bremen, Germany; 4 Netherlands Interdisciplinary Demographic Institute, The Hague, The Netherlands

**Keywords:** Health transitions, Migration, Ageing, Europe

## Abstract

**Background:**

Most previous research on migrant health in Europe has taken a cross-sectional perspective, without a specific focus on the older population. Having knowledge about inequalities in health transitions over the life course between migrants and non-migrants, including at older ages, is crucial for the tailoring of policies to the demands of an ageing and culturally diverse society. We analyse differences in health transitions between migrants and non-migrants, specifically focusing on the older population in Europe.

**Methods:**

We used longitudinal data on migrants and non-migrants aged 50 and older in 10 southern and western European countries from the Survey of Health, Ageing and Retirement in Europe (2004–2015). We applied multinomial logistic regression models of experiencing health deterioration among individuals in good health at baseline, and of experiencing health improvement among individuals in poor health at baseline, separately by sex, in which migrant status (non-migrant, western migrant, non-western migrant) was the main explanatory variable. We considered three dimensions of health, namely self-rated health, depression and diabetes.

**Results:**

At older ages, migrants in Europe were at higher risk than non-migrants of experiencing a deterioration in health relative to remaining in a given state of self-rated health. Western migrants had a higher risk than non-migrants of becoming depressed, while non-western migrants had a higher risk of acquiring diabetes. Among females only, migrants also tended to be at lower risk than non-migrants of experiencing an improvement in both overall and mental health. Differences in the health transition patterns of older migrants and non-migrants remained robust to the inclusion of several covariates, including education, job status and health-related behaviours.

**Conclusions:**

Our findings indicate that, in addition to having a health disadvantage at baseline, older migrants in Europe were more likely than older non-migrants to have experienced a deterioration in health over the study period. These results raise concerns about whether migrants in Europe are as likely as non-migrants to age in good health. We recommend that policies aiming to promote healthy ageing specifically address the health needs of the migrant population, thereby distinguishing migrants from different backgrounds.

**Electronic supplementary material:**

The online version of this article (10.1186/s12916-018-1044-4) contains supplementary material, which is available to authorized users.

## Background

As European societies become older and more diverse [1], the study of the health of older migrants in Europe is becoming increasingly relevant. Having detailed knowledge on how health transitions differ between migrants and non-migrants over the life course is crucial in assessing the future healthcare demands of a society that is becoming older and more culturally diverse [2]. Having such knowledge is also helpful for policymakers who are attempting to adapt their interventions to achieve health equity, which is one of the main pillars of European healthcare systems and policies [3].

Most of the previous research on older migrants’ health in Europe has taken a cross-sectional perspective. These studies showed that, regardless of a generally lower socioeconomic status, migrants tend to live longer than non-migrants; this so-called ‘migrant mortality paradox’ has been observed across the life course, including at older ages [4, 5]. However, previous research has also acknowledged that, compared to non-migrants, older migrants in Europe can expect to live a smaller number of years and a smaller share of their remaining life expectancy in good health [6]. Indeed, compared to older non-migrants, older migrants in Europe tend to have poorer self-rated health, more chronic conditions, worse functioning and higher rates of depression [4, 6–9]. Longitudinal studies can provide a more complete picture than cross-sectional studies of how health and health inequalities evolve over the life course of individuals, and can provide valuable information about the causes of such inequalities.

Several studies have investigated the health differences between migrants and non-migrants in a longitudinal manner [10–16]. These studies have found that migrants, who often have a health advantage relative to non-migrants at arrival, tend to experience steeper rates of health decline with age and length of stay; thus, the health status of migrants tends to converge with that of non-migrants. However, only two of these previous studies specifically focused on the older population [14, 16]. A specific focus on the older population is essential for gaining a better understanding of healthy ageing in a multicultural context, the implications of which vary from maintaining the ability to work at older working ages, which is in itself a protective health factor, to increased quality of life and the possibility to live independently at advanced ages [17].

Furthermore, all of the abovementioned studies examining how the health transitions of migrants and non-migrants differ have been focused on the United States of America (USA) or Canada; yet, whether their findings are also valid in a European context remains unclear. A majority of the older migrants who currently live in western Europe arrived before the early 1970s as labour migrants, or from neighbouring countries or former colonies [1]. We know that many years after migration, older migrants in Europe tend to be disadvantaged relative to non-migrants in terms of self-rated health, chronic conditions, functioning, limitations and depression [4, 6–9]. This is an important difference with respect to the USA and Canada, where older migrants have been shown to have an overall health advantage relative to non-migrants at baseline [14, 16]. On the one hand, this implies that, in Europe, the migrant health advantage at the time of arrival disappears by the time migrants have reached age 50. On the other hand, if migrants in Europe were to maintain steeper rates of health decline than non-migrants at older ages, this would inevitably lead to an increase of migrant health inequalities.

To our knowledge, only a single study so far has described how the health transition patterns of older migrants and non-migrants in Europe differ [18], focusing on the extent to which these two groups maintained good health and experienced health recovery. The authors found that, as compared to non-migrants, older migrants had a lower probability to remain in good health, and a lower probability to experience an improvement in health. However, their paper did not consider other health variables besides self-rated health, and they did not attempt to explain the differences in health transitions between older migrants and non-migrants based on their demographic, socioeconomic or lifestyle-related characteristics.

Moreover, previous studies on the differences in the health transition patterns of older migrants and non-migrants either did not distinguish migrants according to their place of origin [16], or focused on very specific origin groups, such as Hispanic [14] or eastern European [18]. The specific origin of migrants is likely to play an important role in determining differences in health transitions relative to non-migrants. For example, the health status of migrants at the time of arrival is determined to a large extent by the physical, socioeconomic and political environment of their country or area of origin [19]. In addition, the context of origin may affect migrant health transition patterns at older ages, since specific diseases that tend to develop later in life, such as stomach cancer, may be associated with deprivation during childhood [19].

The aim of the present longitudinal study is to analyse the differences in the health transition patterns of migrants and non-migrants, specifically focusing on the older population in Europe, and to illustrate how a range of individual health determinants contribute to explaining these differences in health transition patterns. In our analysis, we incorporate three dimensions of health, namely a subjective measure of overall health (self-rated health), a measure of mental health (depression) and a measure of physical health (diabetes). As in previous migrant health research [6, 20–23], we also distinguish between western and non-western migrants.

## Methods

### Setting

Our study population consisted of individuals aged 50 and older who participated in the Survey of Health, Ageing and Retirement in Europe (SHARE). Research on individuals aged 50 and older is common in the literature on health at older ages [4–9, 14], and starting from this relatively young age enabled us to study not only health deterioration but also health improvement (which is less common at more advanced ages).

Since 2004, SHARE has been collecting panel data on the health status, the socioeconomic status and the social networks of older individuals in European countries and Israel [24]. For our analysis, we selected data from countries in western and southern Europe only, namely Austria, Belgium, Denmark, France, Germany, Italy, the Netherlands, Spain, Sweden and Switzerland. We excluded eastern European countries because they have very different migration histories than western European countries, with most remaining mainly emigration countries [25]. We used data from waves 1 (2004–2005), 2 (2006–2007), 4 (2011–2012), 5 (2013) and 6 (2015) [26–30]. At each wave, refreshment samples were drawn to increase the sample size and to compensate for panel attrition [24]. We included respondents in wave 1 and in the successive refreshment samples for whom health data were available for at least two waves. Data from wave 6 were not available for the Netherlands, leading to a greater proportion of transitions ending in attrition for this country. Results from a sensitivity analysis excluding the Netherlands from the data remained in the same direction, although occasionally an effect lost statistical significance.

### Dependent variable

We defined health transitions (see analysis below), our dependent variable, based on health status at baseline and follow-up. Although self-rated health is often dichotomised into good or more and less-than-good (e.g. [10]), this might conceal certain transition patterns to and from fair health. A recent study showed that the variations in self-rated health response patterns are not strongly related to migrant origin, but rather to the survey language [31]. SHARE questionnaires are provided in the national languages only, which helps reduce the potential variability in the response patterns of migrants versus non-migrants within each country. However, the likelihood of assessing one’s health in a certain way might differ between countries, especially because the term ‘fair’ has distinct connotations in different languages [31]. Moreover, although the validity of self-rated health is well documented in cross-sectional research, reported changes in self-rated health over time might be caused by changes in expectations or in the awareness of health problems [32]. We therefore considered an additional measure of mental health (depression), and an additional measure of physical health (diabetes).

Answers to the question: “Would you say your health is…?” (originally in five categories) were recoded into three categories, i.e. as indicating good (excellent, very good or good), fair or poor self-rated health. Depression was measured using the EURO-D scale [33], which consists of 12 items, namely depression, pessimism, death wish, guilt, sleep, interest, irritability, appetite, fatigue, concentration, enjoyment and tearfulness. Individuals with a EURO-D scale score of more than three were classified as suffering from depression [34]. Respondents who answered “yes” to the question: “Has a doctor ever told you that you had any diabetes or high blood sugar?” were considered to have diabetes.

We converted the data into a person-wave format, allowing as many person-wave observations (health status at baseline combined with health status at follow-up) as possible per respondent. In order to minimise the number of observations ending in loss to follow-up, we also included observations from non-consecutive waves when health information was missing in intermediate waves. Observations from non-consecutive waves represented 3–5% of all observations among non-migrants, western migrants and non-western migrants, and occurred more often among younger, less educated and non-retired respondents. We took into account the differential time of exposure in different transitions by including the pairs of waves as a control variable (see below).

The analytical sample for the analysis of self-rated health consisted of 66,660 respondents who contributed 127,136 person-wave observations. Of these, 116,537 corresponded to non-migrants, 7854 to western migrants and 2745 to non-western migrants. Because a given respondent may provide an answer to one health question but not to another, the samples for analysing depression (*n* = 124,167) and diabetes (*n* = 127,042) were slightly different.

### Independent variables

We defined migrants, our main independent variable, as those respondents who were not born in their current country of residence. As in previous migrant health research [6, 20–23], we distinguished between migrants with a western or non-western origin. We defined western migrants as those born in Europe (except Turkey), North America, Oceania or Japan [6, 23]. Data restrictions did not allow us to distinguish more specific categories of migrant origins, nor motives for migration. The distinction between western and non-western migrants allowed us to account for the role of the context of origin. The environmental, socioeconomic and political context in the country of origin of migrants has an important role in determining their baseline health status and this is especially relevant when the countries involved can be positioned in different stages of the epidemiologic transition [19]. Moreover, the culture and behaviours of non-European migrants are more distant from those of the host society [35]. The vast majority of western migrants in our data (98%) were of European origin. The majority of non-western migrants had been born in one of the following five countries, namely Morocco, Algeria, Turkey, Indonesia and Congo.

We included age to adjust for different age structures in the migrant and non-migrant populations, and country of residence and wave to adjust for contextual differences across space and time. We additionally adjusted for other factors known to be related to health. Being married or partnered is associated with better health outcomes [36]. Poor socioeconomic status is strongly associated with both poor physical and poor mental health outcomes [32, 37, 38]. While job status captures an individual’s current socioeconomic status, level of education also partially reflects socioeconomic position during childhood and youth [39]. Health-related behaviours, and particularly body mass index (BMI), exercise habits and smoking history, are all strongly associated with health outcomes [40].

All covariates, except for an indicator for the pair of waves to which the observation pertained, were measured at the initial wave of each observation (baseline), namely age, country of residence and length of residence in that country, marital status, socioeconomic status (education, job status) and health-related behaviours (BMI, smoking, physical activity). Age was recoded into 5-year age groups up to 85+. Length of residence (up to 10 years and 10 years or longer) was derived from the year of migration and the year when the interview took place; this distinction was also used in a previous study [23] to demonstrate that the initial healthy migrant effect wears off with increasing length of stay, effectively finding differences in the health status of migrants relative to non-migrants according to the length of residence in the country. Distinguishing shorter periods was not feasible because 94% of older migrants in our data had been living in the countries of destination for more than 10 years. We coded marital status into four categories as married^1^ (consisting of the categories “married and living together with spouse” and “registered partnership”), separated (consisting of the categories “married and living separated from spouse” and “divorced”), single (“never married”) and widowed. International Standard Classification of Education 1997 codes of highest level of education were recoded into four categories as primary education or lower (codes 0 and 1), secondary education (codes 2 and 3), higher education (codes 4, 5 and 6), and other (consisting of the categories “still in education” and “other”). Current job status was recoded into four categories as retired, economically active (“employed” or “self-employed”), unemployed or economically inactive (“unemployed”, “permanently sick or disabled” or “homemaker”) and other. We used the original BMI coding of underweight (< 18.5), normal weight (18.5–24.9), overweight (25–29.9) and obese (> 30). We also maintained the dichotomous coding for ever having smoked (yes/no), and the four categories indicating how frequently the respondent engaged in either vigorous or moderate activities as more than once a week, once a week, one to three times a month, and hardly ever or never. In SHARE, vigorous activities are defined as sports, heavy housework and physically demanding jobs, while moderate activities include less demanding forms of exercise such as gardening, cleaning the car or going for a walk.

### Statistical analysis

We performed two-sided tests to assess whether the differences in the background characteristics and the health status at baseline between older migrants (western and non-western) and non-migrants were statistically significant.

We applied multinomial logistic regression models separately by sex. While other methods, such as ordered logistic regression models, would have allowed to retain the original five-category self-rated health variable, such methods would not allow distinguishing health deterioration from health improvement. More importantly, we would have to exclude observations ending in attrition, which would potentially bias our findings.

**Fig. 1:**
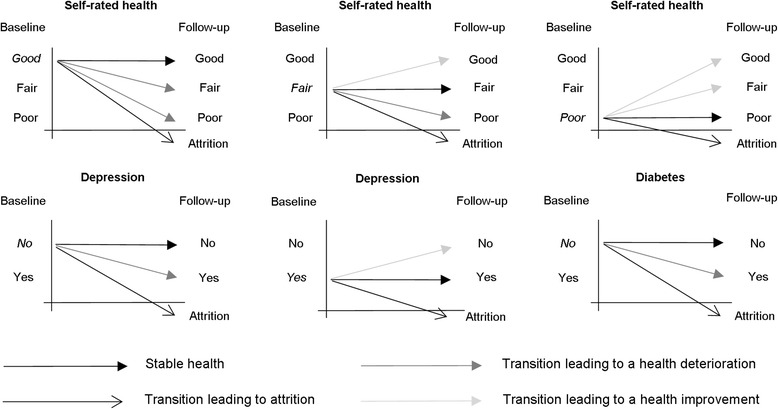
Definition of transitions based on health at baseline and follow-up

We ran separate models for transitions starting in good, fair and poor self-rated health, since the possible health transitions were restricted by the health status at baseline. Compared to the reference category (remaining in the same health status), those initially in good health could experience transitions leading to health deterioration (to fair health or to poor health) or to attrition (either to death or to loss to follow-up). Those initially in fair health could experience health improvement (to good health), health deterioration (to poor health) or be lost to attrition. Those initially in poor health could experience health improvement (to fair health or to good health) or be lost to attrition (Fig. 1).

Similarly, we ran separate models for those transitions starting in a non-depressed state, and for those transitions starting in a depressed state. Compared to remaining non-depressed, the possible transitions were experiencing health deterioration (becoming depressed), and being lost to attrition. Likewise, compared to remaining depressed, the possible transitions were experiencing health improvement (recovering from depression) and being lost to attrition.

For diabetes, we only considered transitions starting in a healthy state (non-diabetic), because recovery is unlikely, although healthier lifestyles have been shown to mitigate its negative effects on health (e.g. [41]). Compared to remaining in a healthy state (non-diabetic), the possible transitions were experiencing health deterioration (becoming diabetic) and being lost to attrition.

Unfortunately, the data did not allow us to differentiate transitions leading to death from those leading to loss to follow-up. However, we modelled transitions leading to attrition (either death or loss to follow-up) as a competing risk in each of the analyses. This is important because, unfortunately, attrition cannot be seen as random since death is obviously a health outcome and loss to follow-up may also be related to health problems.

We estimated robust standard errors [42, 43] to take into account the fact that the same respondent may be observed several times (transition or no transition). Models were run in three steps. In step 1, we included migrant origin (non-migrant, western migrant or non-western migrant) and controlled for age, country of residence and wave. In step 2, we additionally controlled for each respondent’s length of residence in the country, marital status, highest level of education attained and current job status. Finally, in step 3, we additionally controlled for BMI, having ever smoked and frequency of engaging in vigorous and moderate activities.

## Results

### Descriptive findings

Table 1 shows the absolute and the relative distributions of the person-wave observations according to individual characteristics at baseline by migrant origin, for the sample used in the analysis of self-rated health deriving from data on older respondents (aged 50 and older) in 10 southern and western European countries in SHARE (2004–2015). Compared to non-migrants, the proportion of males was lower among western migrants, but higher among non-western migrants. While western migrants had a similar age profile to that of non-migrants, non-western migrants tended to be younger. The vast majority of migrants had been living in the current country of residence for more than 10 years. Compared to non-migrants, all migrants were more likely to be separated and less likely to be married, non-western migrants were less likely to be widowed and western migrants were less likely to be single. While larger shares of both western and non-western migrants than of non-migrants were highly educated, the share of non-western migrants with primary education or lower was also larger. In line with their younger age structure, non-western migrants were less likely than non-migrants to be retired, and more likely to be economically active, unemployed or economically inactive, whereas the job status profile of western migrants was very similar to that of non-migrants. Non-western migrants were less likely than both non-migrants and western migrants to report frequently engaging in vigorous and moderate activities. However, in terms of smoking and BMI, non-western migrants had a slightly healthier profile, as the shares of non-western migrants who had ever smoked or were overweight were smaller than those of both non-migrants and western migrants.

**Table 1:** Person-wave observations^a^ according to individual characteristics at baseline by migrant origin (2004–2015)

	Non-migrants		Western migrants		Non-western migrants	
	*N*	%	*N*	%	*N*	%
Sex						
Male	53,353	45.8	3267^*^	42.8^*^	1482^*^	50.0^*^
Female	63,184	54.2	4370^*^	57.2^*^	1480^*^	50.0^*^
Age, years						
50–54	24,258	20.8	1590	20.8	1052^*^	35.5^*^
55–59	22,012	18.9	1336^*^	17.5^*^	635^*^	21.4^*^
60–64	20,125	17.3	1344	17.6	465	15.7
65–69	16,994	14.6	1202^*^	15.7^*^	345^*^	11.6^*^
70–74	13,860	11.9	911	11.9	223^*^	7.5^*^
75–79	10,070	8.6	655	8.6	137^*^	4.6^*^
80–84	5875	5.0	409	5.4	75^*^	2.5^*^
85+	3343	2.9	190	2.5	30^*^	1.0^*^
Length of residence (years)						
0–9	0	0.0	450	5.9	191	6.5
10+	116,537	100.0	7172	94.1	2763	93.5
Marital status						
Married	85,089	73.1	5353^*^	70.1^*^	2095^*^	70.8^*^
Separated	10,092	8.7	898^*^	11.8^*^	406^*^	13.7^*^
Single	7010	6.0	396^*^	5.2^*^	173	5.8
Widowed	14,285	12.3	989	13.0	287^*^	9.7^*^
Education						
Primary or lower	32,385	27.8	1385^*^	18.2^*^	894^*^	30.3^*^
Secondary	55,767	47.9	3467^*^	45.4^*^	1100^*^	37.3^*^
Higher	27,773	23.8	2614^*^	34.3^*^	879^*^	29.8^*^
Other	536	0.5	163^*^	2.1^*^	74^*^	2.5^*^
Job status						
Retired	54,888	47.4	3664	48.3	874^*^	29.7^*^
Active	37,019	31.9	2412	31.8	1124^*^	38.3^*^
Unemployed or inactive	22,472	19.4	1418	18.7	872^*^	29.7^*^
Other	1537	1.3	89	1.2	68^*^	2.3^*^
BMI						
Underweight	1365	1.2	123^*^	1.6^*^	37	1.3
Normal weight	45,393	39.8	2897	38.6	1213	42.0
Overweight	47,382	41.6	3198	42.6	1107^*^	38.3^*^
Obese	19,807	17.4	1281	17.1	530	18.4
Ever smoked						
No	60,766	52.3	3929	51.7	1656^*^	56.1^*^
Yes	55,433	47.7	3671	48.3	1294^*^	43.9^*^
Vigorous activities						
More than once a week	43,078	37.1	2851	37.5	936^*^	31.8^*^
Once a week	15,866	13.7	1022	13.5	345^*^	11.7^*^
One to three times a month	9538	8.2	540^*^	7.1^*^	200^*^	6.8^*^
Hardly ever or never	47,700	41.1	3185	41.9	1466^*^	49.7^*^
Moderate activities						
More than once a week	83,372	71.8	5578^*^	73.4^*^	1909^*^	64.7^*^
Once a week	14,959	12.9	925	12.2	466^*^	15.8^*^
One to three times a month	5806	5.0	356	4.7	153	5.2
Hardly ever or never	12,053	10.4	739	9.7	423^*^	14.3^*^
TOTAL	116,537		7637		2962	

Source: Own calculations based on data from respondents aged 50 and older in 10 southern and western European countries in SHARE (2004–2015)

^*^Proportion statistically significantly different from that of non-migrants (*p* < 0.05), except for length of residence (difference between western and non-western migrants)

^a^These observations pertain to the sample used in the analysis of self-rated health transitions (see methods section for information on the various samples). The comparison of the background characteristics of the person-wave observations of migrants and non-migrants in the samples used in the analyses of depression and diabetes followed the same pattern

Table 2 shows the counts and the proportions of the different categories of the person-wave observations according to health by sex and migrant origin. At baseline, older western migrants had worse self-rated health outcomes than older non-migrants. Although the self-rated health of non-western migrants at baseline did not seem to differ from that of non-migrants, non-western migrants were more likely to report diabetes or depression. Migrants, especially those of non-western origin, were more likely to have made transitions leading to death or loss to follow-up, and less likely to have experienced no transition (stable good health, stable poor health) than non-migrants. These patterns were present among both males and females. Among females only, compared to non-migrants, western migrants were less likely to experience an improvement in self-rated health and non-western migrants were less likely to recover from depression.

**Table 2:** Person-wave observations according to health at baseline and follow-up by sex and migrant origin (2004–2015)

Males	Non-migrants		Western migrants		Non-western migrants	
	*N*	%	*N*	%	*N*	%
TOTAL: good health^a^ (baseline)	37,241	69.8	2183^*^	66.8^*^	1013	68.4
Stable good health	23,541	63.2	1287^*^	59.0^*^	553^*^	54.6^*^
Health deterioration	4933	13.2	299	13.7	129	12.7
Good health to death/loss to follow-up	8767	23.5	597^*^	27.3^*^	331^*^	32.7^*^
TOTAL: poor health (baseline)	16,112	30.2	1084^*^	33.2^*^	469	31.6
Stable poor health	7573	47.0	466^*^	43.0^*^	167^*^	35.6^*^
Health improvement	3288	20.4	190	17.5	92	19.6
Poor health to death/loss to follow-up	5251	32.6	428^*^	39.5^*^	210^*^	44.8^*^
TOTAL: non-depressed (baseline)	43,159	82.8	2599	81.3	1050^*^	75.3^*^
Stable non-depressed	28,302	65.6	1582^*^	60.9^*^	583^*^	55.5^*^
Health deterioration	3576	8.3	221	8.5	83	7.9
Non-depressed to death/loss to follow-up	11,281	26.1	796^*^	30.6^*^	384^*^	36.6^*^
TOTAL: depressed (baseline)	8941	17.2	598	18.7	344^*^	24.7^*^
Stable depressed	2946	32.9	164^*^	27.4^*^	101	29.4
Health improvement	3014	33.7	203	33.9	99	28.8
Depressed to death/loss to follow-up	2981	33.3	231^*^	38.6^*^	144^*^	41.9^*^
TOTAL: non-diabetic (baseline)	46,858	87.9	2850	87.3	1230^*^	83.2^*^
Stable non-diabetic	33,123	70.7	1869^*^	65.6^*^	739^*^	60.1^*^
Health deterioration	1545	3.3	83	2.9	51	4.1
Non-diabetic to death/loss to follow-up	12,190	26.0	898^*^	31.5^*^	440^*^	35.8^*^
TOTAL: diabetic (baseline)	6461	12.1	413	12.7	248^*^	16.8^*^
Females	Non-migrants		Western migrants		Non-western migrants	
	*N*	%	*N*	%	*N*	%
TOTAL: good health^a^ (baseline)	41,424	65.6	2682^*^	61.4^*^	928	62.7
Stable good health	25,887	62.5	1600^*^	59.7^*^	514^*^	55.4^*^
Health deterioration	6016	14.5	383	14.3	123	13.3
Good health to death/loss to follow-up	9521	23.0	699^*^	26.1^*^	291^*^	31.4^*^
TOTAL: poor health (baseline)	21,760	34.4	1688^*^	38.6^*^	552	37.3
Stable poor health	11,338	52.1	863	51.1	242^*^	43.8^*^
Health improvement	4293	19.7	264^*^	15.6^*^	90	16.3
Poor health to death/loss to follow-up	6129	28.2	561^*^	33.2^*^	220^*^	39.9^*^
TOTAL: non-depressed (baseline)	42,621	68.9	2794^*^	65.6^*^	830^*^	59.1^*^
Stable non-depressed	25,932	60.8	1589^*^	56.9^*^	441^*^	53.1^*^
Health deterioration	6057	14.2	389	13.9	111	13.4
Non-depressed to death/loss to follow-up	10,632	24.9	816^*^	29.2^*^	278^*^	33.5^*^
TOTAL: depressed (baseline)	19,194	31.1	1463^*^	34.4^*^	574^*^	40.9^*^
Stable depressed	8129	42.4	609	41.6	236	41.1
Health improvement	5712	29.8	401	27.4	119^*^	20.7^*^
Depressed to death/loss to follow-up	5353	27.9	453^*^	31.0^*^	219^*^	38.2^*^
TOTAL: non-diabetic (baseline)	56,917	90.2	3923	89.8	1294^*^	87.5^*^
Stable non-diabetic	41,465	72.9	2717^*^	69.3^*^	820^*^	63.4^*^
Health deterioration	1460	2.6	100	2.5	40	3.1
Non-diabetic to death/loss to follow-up	13,992	24.6	1106^*^	28.2^*^	434^*^	33.5^*^
TOTAL: diabetic (baseline)	6218	9.8	445	10.2	185^*^	12.5^*^

Source: Own calculations based on data from respondents aged 50 and older in 10 southern and western European countries in SHARE (2004–2015)

^*^Proportion statistically significantly different from that of non-migrants (*p* < 0.05)

^a^The category “good” self-rated health consists of the original categories “excellent”, “very good” and “good”. Additional analyses revealed that the proportions of transitions within these three original states did not differ between older migrants and non-migrants

### Health deterioration

The differences between older migrants and older non-migrants in the likelihood of experiencing health deterioration were robust to the inclusion of a wide variety of covariates that are strongly associated with health outcomes and transitions (socioeconomic status, health-related behaviours, marital status). We therefore show only the coefficients for the fully adjusted model. The complete results, including all steps and the effects of all covariates, as well as the models showing the risk of experiencing transitions leading to attrition (death or loss to follow-up) are shown in the appendix (Additional file 1).

Table 3 shows the risk of experiencing a transition relative to remaining in a given state of self-rated health on the logit scale, by sex. We found that, among those initially in good self-rated health, both older western and older non-western migrants faced a higher risk than older non-migrants of deteriorating health as compared to maintaining good health. The effect of being a migrant on health deterioration tended to be stronger for transitions leading to poorer states of health. That is, the difference in the risk of experiencing a transition between migrants and non-migrants was higher for transitions leading from good to poor health than for transitions leading from good to fair health. These patterns were found for both sexes, although the differences in the risk of health deterioration between non-western female migrants and female non-migrants were not statistically significant. Among females only, migrants had a higher risk of experiencing health deterioration as compared to remaining in fair health, while the risk of transitioning from fair to poor health did not seem to differ much between male migrants and male non-migrants.

**Table 3:** Effects^a^ (logit) of experiencing a transition in self-rated health, by sex (2004–2015)

Effects (logit) of transitioning as compared to remaining in good (or more) self-rated health								
	Males (*N* = 39,807)				Females (*N* = 43,839)			
Log pseudo-likelihood	−35,304				−38,987			
Pseudo R^2^	0.0735				0.0820			
	to fair health		to poor health		to fair health		to poor health	
	b	SE	b	SE	b	SE	b	SE
Origin: non-migrants (ref)								
Western migrants	0.19**	0.08	0.37**	0.16	0.21***	0.07	0.29*	0.16
Non-western migrants	0.20*	0.12	0.45*	0.25	0.19	0.12	0.21	0.26
Effects (logit) of transitioning as compared to remaining in fair self-rated health								
	Males (N = 12,763)				Females (*N* = 16,944)			
Log pseudo-likelihood	−15,648				−20,737			
Pseudo R^2^	0.0606				0.0583			
	to good health		to poor health		to good health		to poor health	
	b	SE	b	SE	b	SE	b	SE
Origin: non-migrants (ref)								
Western migrants	0.03	0.12	0.02	0.15	−0.20**	0.09	0.26**	0.12
Non-western migrants	0.14	0.18	−0.24	0.25	0.19	0.16	0.41**	0.20
Effects (logit) of transitioning as compared to remaining in poor self-rated health								
	Males (*N* = 4430)				Females (*N* = 5850)			
Log pseudo-likelihood	− 5187				− 6892			
Pseudo R^2^	0.0676				0.0746			
	to good health		to fair health		to good health		to fair health	
	b	SE	b	SE	b	SE	b	SE
Origin: non-migrants (ref)								
Western migrants	0.23	0.25	−0.10	0.19	−0.31	0.24	− 0.35**	0.16
Non-western migrants	0.91**	0.40	0.16	0.35	−0.07	0.40	−0.09	0.27

Source: Own calculations based on data from respondents aged 50 and older in 10 southern and western European countries in SHARE (2004–2015)

^a^The effects shown pertain to the fully adjusted model. Results for the intermediate steps are shown in the appendix

* *p* < 0.1, ** *p* < 0.05, *** *p* < 0.01

Table 4 shows the risk of experiencing a transition in mental health (depression) as compared to remaining in a given state of health. For both sexes, older western migrants had a higher risk than older non-migrants of becoming depressed as compared to remaining non-depressed. The risk of becoming depressed did not seem to differ between non-western migrants and non-migrants.

**Table 4:** Effects^a^ (logit) of experiencing a transition in mental health, by sex (2004–2015)

Effects (logit) of becoming depressed as compared to remaining non-depressed				
	Males (*N* = 46,137)		Females (*N* = 45,076)	
Log pseudo-likelihood	−36,011		−38,987	
Pseudo R^2^	0.0673		0.0666	
	b	SE	b	SE
Origin: non-migrants (ref)				
Western migrants	0.17**	0.08	0.15**	0.07
Non-western migrants	0.02	0.13	0.00	0.12
Effects (logit) of recovering from depression as compared to remaining depressed				
	Males (*N* = 9612)		Females (*N* = 20,249)	
Log pseudo-likelihood	− 9788		−20,395	
Pseudo R^2^	0.0730		0.0676	
	b	SE	b	SE
Origin: non-migrants (ref)				
Western migrants	0.15	0.12	−0.24***	0.08
Non-western migrants	0.01	0.16	−0.37***	0.13

Source: Own calculations based on data from respondents aged 50 and older in 10 southern and western European countries in SHARE (2004–2015)

^a^The effects shown pertain to the fully adjusted model. Results for the intermediate steps are shown in the appendix

** *p* < 0.05, *** *p* < 0.01

Table 5 shows the risk of becoming diabetic as compared to remaining non-diabetic. The risk of acquiring diabetes was substantially higher for older non-western migrants than for older non-migrants. Western female migrants were also at a higher risk of becoming diabetic than female non-migrants.

**Table 5:** Effects^a^ (logit) of becoming diabetic as compared to remaining non-diabetic, by sex (2004–2015)

	Males (*N* = 50,001)		Females (*N* = 60,094)	
Log pseudo-likelihood	−32,819		−36,874	
Pseudo R^2^	0.0766		0.0834	
	b	SE	b	SE
Origin: non-migrants (ref)				
Western migrants	0.10	0.12	0.25**	0.12
Non-western migrants	0.56***	0.17	0.43**	0.19

Source: Own calculations based on data from respondents aged 50 and older in 10 southern and western European countries in SHARE (2004–2015)

^a^The effects shown pertain to the fully adjusted model. Results for the intermediate steps are shown in the appendix

** *p* < 0.05, *** *p* < 0.01

The effects of all covariates on the risk of health deterioration were rather similar regardless of the specific dimension of health considered (self-rated health, depression or diabetes) (Additional file 1). The risk of health deterioration increased with age. Lower socioeconomic status and risky health-related behaviours were associated with a greater likelihood of experiencing transitions leading to poorer health outcomes. The risk of health deterioration decreased with increasing levels of education. Respondents who were economically active were less likely than those who were retired to have experienced health deterioration. Being unemployed or economically inactive was associated with a higher risk of health deterioration than being retired, especially among males. The effect of the socioeconomic covariates on the risk of health deterioration remained similar after adjusting for health behaviours. Being underweight, overweight or obese, having ever smoked, and exercising less frequently were all factors that substantially increased the risk of transitioning to a poorer health status.

### Health improvement

In general, the risk of experiencing an improvement in health as compared to remaining in a given state of self-rated health for older migrants did not seem to differ from that of older non-migrants (Table 3). Western female migrants were less likely than female non-migrants to have experienced an improvement from poor to fair, and from fair to good self-rated health as compared to maintaining poor or fair health, respectively. Non-western male migrants tended to be more likely than male non-migrants to have experienced an improvement from poor to good health as compared to maintaining poor health.

Older female migrants, and especially those of non-western origin, were less likely than older female non-migrants of experiencing a recovery from depression as compared to remaining depressed (Table 4). Among males, the risk of recovering from depression as compared to remaining depressed did not seem to differ between migrants and non-migrants.

The effects on the risk of experiencing an improvement in health among older migrants and non-migrants remained very similar in size and in the same direction after all of the covariates had been included in the analysis (Additional file 1). The likelihood of health improvement decreased with age. Single and separated respondents were less likely to have experienced an improvement in health than married respondents. The risk of health improvement was lower among those with primary education or lower than among those with secondary education. Respondents who were economically active were more likely than those who were retired to have experienced an improvement in health, while the opposite was the case among respondents who were unemployed or economically inactive. Being underweight, overweight or obese, having ever smoked, and exercising less frequently were associated with a reduced likelihood of recovery. These effects were similar regardless of the dimension of health considered (self-rated health, depression or diabetes). The effect of socioeconomic status on the risk of health improvement remained similar in terms of direction and size after additionally controlling for health behaviours.

## Discussion

### Summary of the results

We applied multinomial regression models to longitudinal data on self-rated health, depression and diabetes derived from the Survey of Health, Ageing and Retirement in Europe (2004–2015) to examine differences in the health transition patterns of migrants and non-migrants aged 50 and older in 10 southern and western European countries. We found that, at older ages, western migrants had poorer self-rated health at baseline than non-migrants, while non-western migrants were more likely than non-migrants to have diabetes or depression. We also found that older migrants in Europe were at higher risk than older non-migrants of experiencing health deterioration as compared to remaining in a given state of self-rated health. Western migrants had a higher risk than non-migrants of becoming depressed, while non-western migrants had a higher risk of acquiring diabetes. Among females only, migrants also tended to be at lower risk than non-migrants of experiencing an improvement in both overall and mental health. Even after the inclusion of several covariates that are strongly associated with health, differences in the health transition patterns of older migrants and non-migrants remained largely unexplained.

### Interpretation of the results

We found that, over the study period, older migrants in Europe were more likely than older non-migrants to have experienced health deterioration and, among females only, less likely to have experienced health improvement. This finding seems to be in line with the steeper rates of health decline among migrants with age and the passage of time, previously observed both at younger adult ages and at older ages [13, 14, 16, 44]. Our results may be explained by the cumulative disadvantage theory [45, 46], which postulates that migrants suffer from the negative effects of having a relatively low socioeconomic position throughout their life course, including detrimental effects on their health. Indeed, migrants often experience material deprivation, poor working conditions, social isolation and limited access to services [47]. Furthermore, failure to meet socioeconomic aspirations, and in particular perceived downward social mobility as compared to the expectations had the person not migrated, can result in an even greater health burden for migrants [48].

Among males only, the risk of experiencing a health improvement did not seem to differ between migrants and non-migrants. Previous research showed that (non-western) migrants tend to suffer more often from infectious diseases and occupational injuries, which, compared to most non-communicable diseases, are more likely to lead to recovery in case of survival [47, 49–51]. It might be possible that recovery from these causes mitigates the negative effects migrants bear due to a general social and economic disadvantage over their life courses. We may speculate that, because of the particularly pronounced gendered social and labour-market position among migrants of mainly non-western origin (e.g. [52]), female migrants might be less prone to occupational injuries and therefore also less likely to recover.

We also found that, at older ages, migrants had poorer health at baseline than non-migrants. This finding is in line with results from previous studies in Europe [4, 6–9]. Although migrants tend to have a health advantage relative to non-migrants at the time of arrival (e.g. [53]), their health tends to decline at a faster pace starting just a few years after arrival [13, 44, 54]. This might explain why, many years after migration, older migrants in Europe tend to be in poorer general health than non-migrants. However, the initial health advantage of migrants does not seem to have reversed by the time they reach old age in the USA or Canada, where steeper rates of health decline among migrants at older ages lead to a decrease in migrant health inequalities and thus to convergence in health between older migrants and non-migrants [14, 16]. In contrast, higher risks of health deterioration and lower risks of health improvement among older migrants in Europe will lead to an increase in migrant health inequalities and thus to divergence in health between older migrants and non-migrants.

Our findings also show that migrant origin plays a role in explaining differences in the health transition patterns of older migrants and non-migrants. The risk of experiencing a deterioration in self-rated health and the risk of acquiring diabetes tended to be higher among older non-western migrants than among older western migrants. In particular, the higher risk of developing diabetes among non-western migrants is likely due to a combination of genetic and physiological factors, conditions during early life such as malnutrition, and potential changes in health-related behaviours after migration [55, 56]. Although the risk of becoming depressed did not differ between non-western migrants and non-migrants, non-western migrants were more likely to have depression than both non-migrants and western migrants at baseline.

However, it was unexpected that non-western male migrants were more likely to experience transitions leading from poor to good self-rated health as compared to remaining in poor health. Given the small sample size of non-western male migrants initially in poor self-rated health (*N* = 120), the effect of outliers in this group might be large. Indeed, 13 non-western male migrants transitioned from poor to good health. These respondents were much younger than non-migrants in this group (below 65), and were more often unemployed or economically inactive. These characteristics did not correspond with the general characteristics of non-western male migrants initially in poor health (results not shown), thereby suggesting that these 13 respondents correspond to cases of acute illness and subsequent recovery.

### Evaluation of data and methods

This study provided new insights into the health transition patterns of older migrants and non-migrants in 10 countries in southern and western Europe by considering measures of overall, mental and physical health. However, some limitations in the data and methods need to be considered.

First, SHARE is not designed to adequately subsample the migrant subpopulation. However, underrepresentation of migrants in our data proved to be only moderate. According to Eurostat data for 2011 [57], migrants represented 9.1% of the population aged 50 and older in the 10 countries studied, whereas in our data based on SHARE, migrants only contributed 8.3% of the person-wave observations. Nevertheless, migrants in the sample are very likely to be selective because SHARE questionnaires are provided in the national languages only. Thus, only migrants who have a good command of the country’s language are eligible. Although the observed pattern of stronger health deterioration among migrants follows the pattern of previous studies outside Europe [10–16], further studies may want to investigate whether our findings can indeed be generalised to the population level. In addition, small migrant sample sizes hampered the classification of migrants beyond a broad western versus non-western typology. Furthermore, the data for all of the countries were pooled together. Although we controlled for the effect of country of residence to broadly consider the spatial, social and institutional context, the impact of this context could well be dissimilar for migrants and non-migrants. For instance, integration policies or public attitudes towards migrants are aspects of the policy and societal context that may affect migrants and their health in particular (e.g. [58]).

Second, due to data restrictions, we were unable to distinguish transitions leading to death from those leading to loss to follow-up. The respondents in SHARE are traced and followed if they relocate within the country, and their mortality is recorded via end-of-life interviews with a proxy respondent, who could be a family or household member, a neighbour or another person socially related to the deceased [24]. A recent study compared the mortality rates in SHARE with those from the Human Mortality Database, and concluded that SHARE underestimates mortality [59]. Although transitions leading to attrition (either death or loss to follow-up) were modelled as a competing risk, the results of this part of the analysis are difficult to interpret. Given that older migrants may have a mortality advantage over older non-migrants in Europe, or at least in certain European countries [4, 5], our results suggest that migrants are more likely to be lost to follow-up than non-migrants. Considering the (rather debated) possibility that older non-migrants in poor health might return to their country of origin, as suggested by the “salmon bias” hypothesis [60], our results may underestimate the relative disadvantage of migrants in transitioning to poorer states of health. Additionally, given that we could not distinguish mortality from losses to follow-up, the relevance of our findings could be challenged by arguing that mortality is the ultimate health outcome. Nevertheless, we argue that general health also matters, and has a clear impact on people’s quality of life [61]. Thus, we believe our findings have implications relevant to health-related policies and healthcare provision.

Third, we could observe a maximum of four person-wave observations per respondent. Respondents first entered the survey in different waves, and not all of the respondents made it to the final wave due to death or loss to follow-up. Because the number of transitions observed per individual was relatively small, we were unable to analyse longer health trajectories. Considerably more effort should be devoted to gathering comparative longitudinal migrant health data across Europe.

Finally, the coefficients for being a migrant might be influenced by an unmeasured residual effect of socioeconomic status on the risk of experiencing a given health transition. Migrants tend to be in a disadvantaged position with respect to non-migrants in a similar socioeconomic position [62]. Yet, the role of socioeconomic status in the health of migrants is complex and not yet well understood; moreover, the various dimensions of socioeconomic status may affect the health of migrants and non-migrants differently [63]. By controlling for the highest level of education and job status only, we may not be able to accurately capture socioeconomic differences between older migrants and non-migrants. Had we been able to include additional control variables indicating socioeconomic status (e.g. income), these might have further explained the inequalities in health transitions between migrants and non-migrants.

## Conclusion

Our study is the first to analyse and explain the differences in the overall, physical and mental health transition patterns of older migrants and non-migrants in a European context. Our results show that older migrants in Europe were more likely than older non-migrants to have experienced health deterioration and, among females only, less likely to have experienced an improvement in health. These patterns were visible for self-rated health, depression and diabetes, and seem to be in line with the social and economic disadvantage migrants tend to experience over their life courses. The transition patterns in terms of depression or diabetes can be thought as examples of how transition patterns in mental and physical health, respectively, shaped transition patterns in overall health. Our results also show that the differences in the health transition patterns of older migrants and non-migrants remained largely unexplained even after a range of socioeconomic indicators and health-related behaviours were taken into account.

Our results raise concerns about whether migrants in Europe are as likely as non-migrants to age in good health, and suggest that general policies aimed at improving health among the older population, such as policies that promote healthier lifestyles or broader socioeconomic policies that seek to tackle socioeconomic inequalities, might not suffice to effectively reduce health inequalities between migrants and non-migrants. We recommend that policies aiming to promote healthy ageing specifically address the health needs of the migrant population, thereby distinguishing migrants from different backgrounds.

Future research should investigate the role of specific diseases and conditions, and the extent to which the context in the country or area of origin and in the country of residence explain the differences in health and health transitions between older migrants and non-migrants. The findings of these studies may, for example, be used to help formulate healthy ageing policies that target specific diseases and conditions that affect migrants in particular, to design more inclusive integration policies and to create campaigns to promote more favourable public attitudes towards migrants.

## Additional file

Additional file 1: Complete results, including all steps and the effects of all covariates, as well as the models showing the risk of experiencing transitions leading to attrition (death or loss to follow-up). (XLSX 87 kb)

## Abbreviations

BMI: body mass index
SHARE: Survey of Health, Ageing and Retirement in Europe
USA: United States of America

## Footnotes

1 Although this is not completely accurate, we use the term “married” to denote both respondents that were married and living with their spouse, and respondents who were in a registered partnership. Registered partnership is a rare category in the data, which was found mainly in Sweden, the Netherlands, and Belgium (5–10% of cases). No information was available on whether those in a registered partnership were actually living with their partner. Neither was information available on informal cohabitation.
